# Achieving single cell acoustic localisation with deactivation super resolution

**DOI:** 10.1038/s44384-025-00008-7

**Published:** 2025-04-24

**Authors:** Cameron A. B. Smith, Mengtong Duan, Jipeng Yan, Laura Taylor, Mikhail Shapiro, Meng-Xing Tang

**Affiliations:** 1 Department of Bioengineering, Imperial College London, London, UK; 2 Division of Chemistry and Chemical Engineering, California Institute of Technology, Pasadena, CA USA; 3 Andrew and Peggy Cherng Department of Medical Engineering, California Institute of Technology, Pasadena, CA USA; 4 Howard Hughes Medical Institute, Pasadena, CA USA

**Keywords:** Biological techniques, Health care, Engineering

## Abstract

Photo-activated localization microscopy (PALM) has been a game-changer, breaking the diffraction limit in spatial resolution. This study presents the Deactivation Super Resolution (DSR) method, which utilises the deactivation of genetically encodable contrast agents, enabling us to super-resolve and pinpoint individual cells with ultrasound as they navigate through structures which cannot be resolved by conventional B-Mode imaging. DSR takes advantage of Gas Vesicles (GVs), which are air-filled sub-micron particles that have been expressed in genetically engineered bacterial and mammalian cells to produce acoustic contrast. Our experimental results show that DSR can distinguish sub-wavelength microstructures that standard B-mode ultrasound images fail to resolve by super-localising individual mammalian cells. This study provides a proof of concept for the potential of DSR to serve as a super-resolution ultrasound technique for individual cell localisation, opening new horizons in the field.

## Introduction

Photo-activated localisation microscopy^1^ has been a great advance in optical imaging, allowing for the breaking of the diffraction limit by utilising activated fluorescent markers to generate super-resolved images. These principles have also been extended to the field of acoustic super-resolution^2^, which is often referred to as super resolution ultrasound or ultrasound localisation microscopy. Here, sparse microbubbles injected into the vasculature can be imaged, localised and tracked to produce super-resolution images of the microvasculature. This technique shows great promise as a tool for cardiovascular^3–6^, neurovascular^7,8^ and tumour imaging^9,10^. In addition, there is potential for applications utilising a wider range of contrast agents, which this work hopes to explore.

Gas vesicles (GVs) are sub-micron air-filled protein-shelled particles^11^. They are cylindrical in shape, with a diameter of ~140 nm and a length of 200–800 nm. GVs are naturally expressed by buoyant photosynthetic microbes^12^ and have recently been shown to be capable of expression in bacterial and mammalian cells as acoustic reporter genes^13–15^. GVs and GV-expressing cells have also been observed extravasating into surrounding tissue^16^, enabling the potential for contrast-enhanced ultrasound imaging throughout the body.

Two methods are predominantly used to image GVs while distinguishing them from the surrounding tissue. The first is based on amplitude modulation^17–19^, which capitalises on the fact that GVs are non-linear oscillators that buckle non-destructively in an acoustic field^20,21^. The second approach leverages the irreversible collapse of GVs under high-pressure pulses through a method called BURST^22^, which transmits a series of high-pressure pulse-echoes. In BURST imaging, the applied pressure is sufficient to collapse GVs in the first high pressure pulse-echo, meaning that the echoes from this pulse contain signals from both GVs and background tissue. However, signals from subsequent pulse-echoes represent only the background tissue. A spectral unmixing technique is then deployed to segregate the GV signal. This effect is amplified by transmitting multiple-cycle pulse trains for a single pulse-echo cycle. This causes the gas released from the GVs to cavitate, producing a strong signal with enhanced contrast when compared to the subsequent frames. Sawyer et al.^22^ found a linear correlation using BURST imaging between the number of bright spots on BURST imaging a beaker of liquid and the cell concentration within that liquid, suggesting that BURST imaging may be capable of detecting individual GV-expressing cells.

In this work, we develop a new imaging technique based on the principles behind BURST. This method enables the imaging of individual cells that highly express GVs and super-localises their positions using a technique we call Deactivation Super Resolution (DSR).

## Results

### Deactivation super resolution

**Fig. 1:**
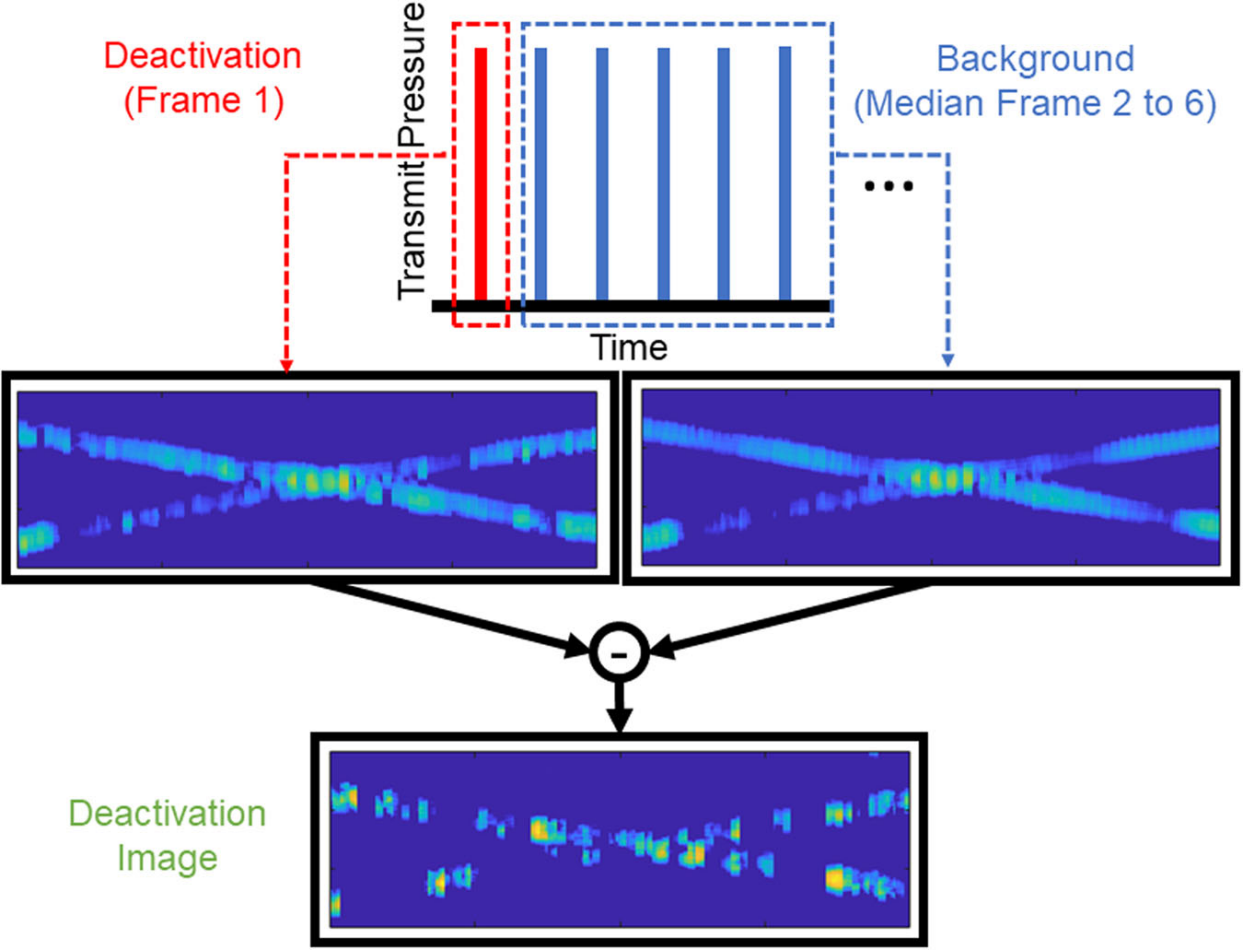
Schematic demonstrating the pulse-echo structure and the processing involved in generating deactivation images.

**Fig. 2:**
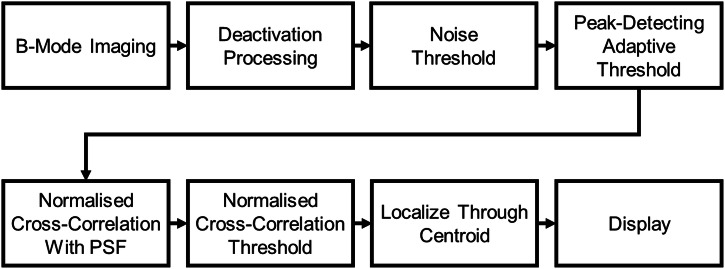
Schematic diagram showing the processing pipeline for deactivation super-resolution.

In the DSR method we use a series of six high pressure (6.2 MPa peak positive pressure) pulses to produce six B-Mode images. At the start of the first of these six pulse-echoes (here called the deactivation pulse) the protein shell of the GV is fractured, releasing the gas within. This gas cavitates, releasing a strong acoustic signal over the remaining cycles, after which the bubble collapses. On the subsequent pulse-echoes the GVs are no longer present, as such the signal that is reflected to the transducers for these pulses represent all the structures in the imaging field except for the GVs. This provides us with an opportunity. By taking the median image of the five images after the deactivation pulse and subtracting this from the deactivation pulse, we end up with a deactivation image which contains the isolated signal from the GVs (Fig. 1). We then use the DSR pipeline to super-localise individual cells (Fig. 2). It is worth noting that 6.2 MPa is greater than the estimated 587 kPa necessary to collapse the GVs^23^, however in previous studies pressures above this value have been found to further increase the signal received by the GVs^22^.

### Super resolved cross tube phantom

**Fig. 3:**
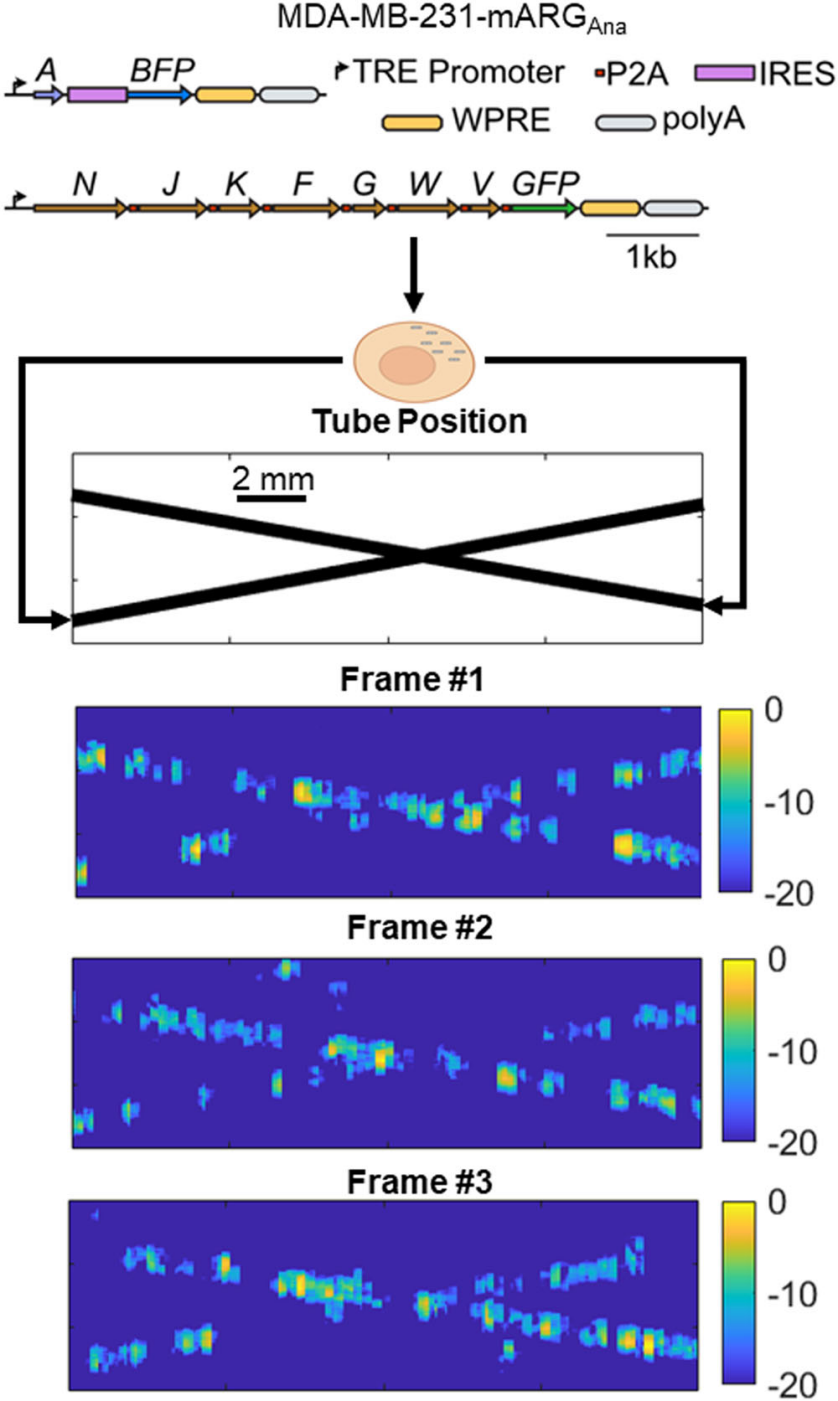
schematic diagram (top) showing the genetically engineered MDA cells (adapted from ref. ^13^) flowing through the tube in set positions, and below representative sample deactivation image frames.

To evaluate the effectiveness of DSR, we used a cross tube phantom^24–26^ which comprises of two thin-walled microvessels of internal diameter 200 μm ± 15 μm arranged in a cross formation. GV-expressing mammalian tumour cells^13^ were flowed through these microvessels using a syringe pump and imaged with our DSR pipeline using an L11-4v probe and using 4 MHz 3 cycle pulse trains. The cells we used were MDA-MB-231-mARG_Ana_ cells as described in Hurt et al.^13^. In brief, MDA-MB-231 cells were engineered to constitutively co-express the plasmids shown in Fig. 3 (top) which enable expression of GVs derived from anabaena, GFP and BFP when induced with the small molecule doxycycline.

**Fig. 4:**
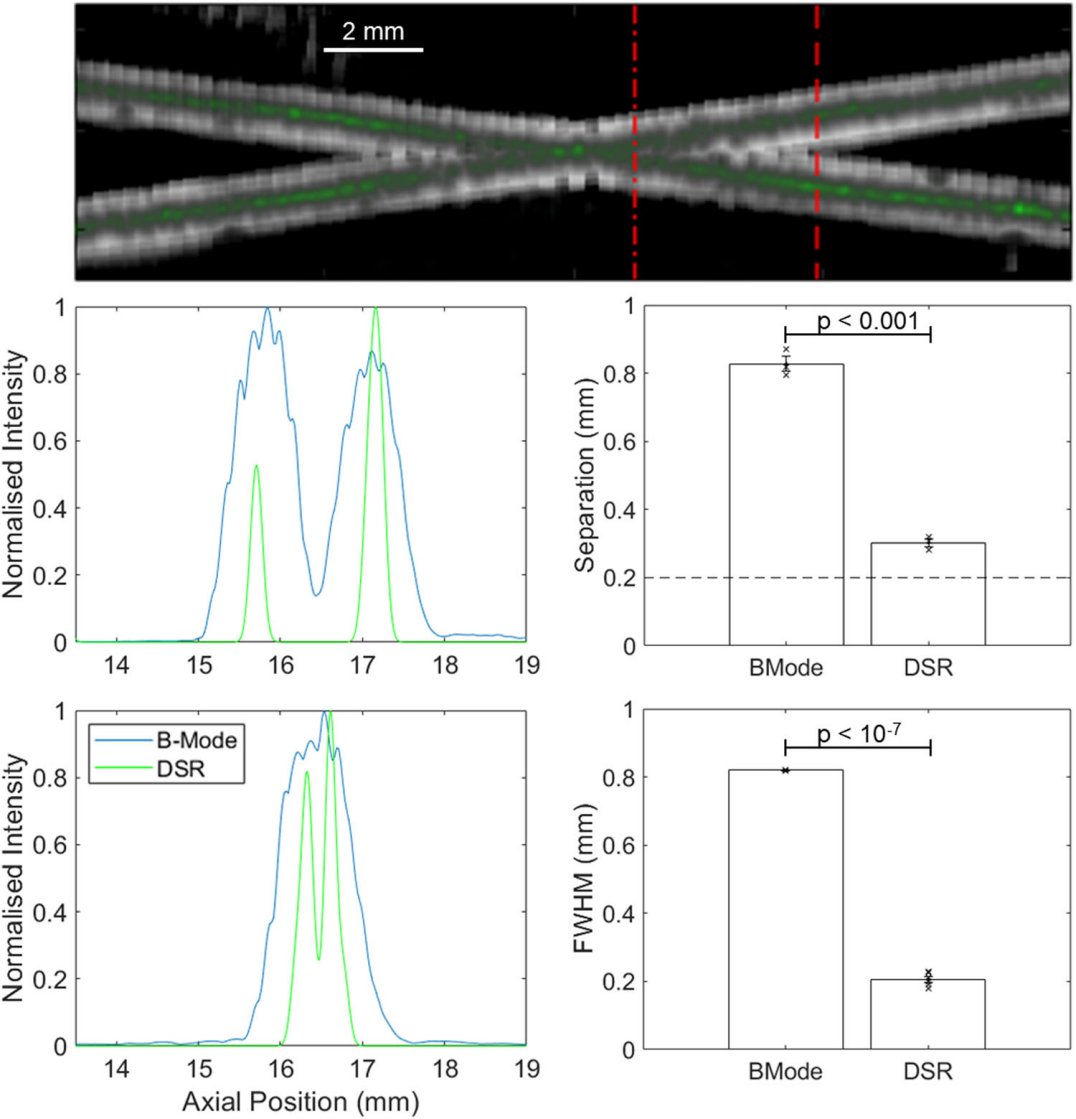
Representative deactivation super resolution image (top) and two cut through line plots showing the separation of the tubes at two positions, bar charts showing the mean and standard error of FWHM (*n* = 6) and tube separation distances (*n* = 3).

Images of the two crossing tubes (Fig. 3) show deactivation frames showing the cells which will later be localised. Final SR images of the phantom show the flow path of cells super-localised to the centre of each channel, resulting in tighter signal distributions across the widths of each tube (Fig. 4). Cross sectional signal profiles reveal that in the first location, where the tubes are further apart, the two tubes are distinguishable on both B-Mode and SR. However, in the second location, where the tubes are closer together, they are only distinguishable in SR. The average separation distance between the two tubes, defined as the closest distance where the two full width at half maximum (FWHM) profiles do not overlap is 0.83 ± 0.01 mm for the B-Mode and 0.30 ± 0.01 mm for the DSR (*n* = 3). The average FWHM for the B-Mode image was 0.82 ± 0.00 mm, while for the SR image it was 0.21 ± 0.01 mm mean ± standard error (*n* = 6).

### Validation of single cell localisation

**Fig. 5:**
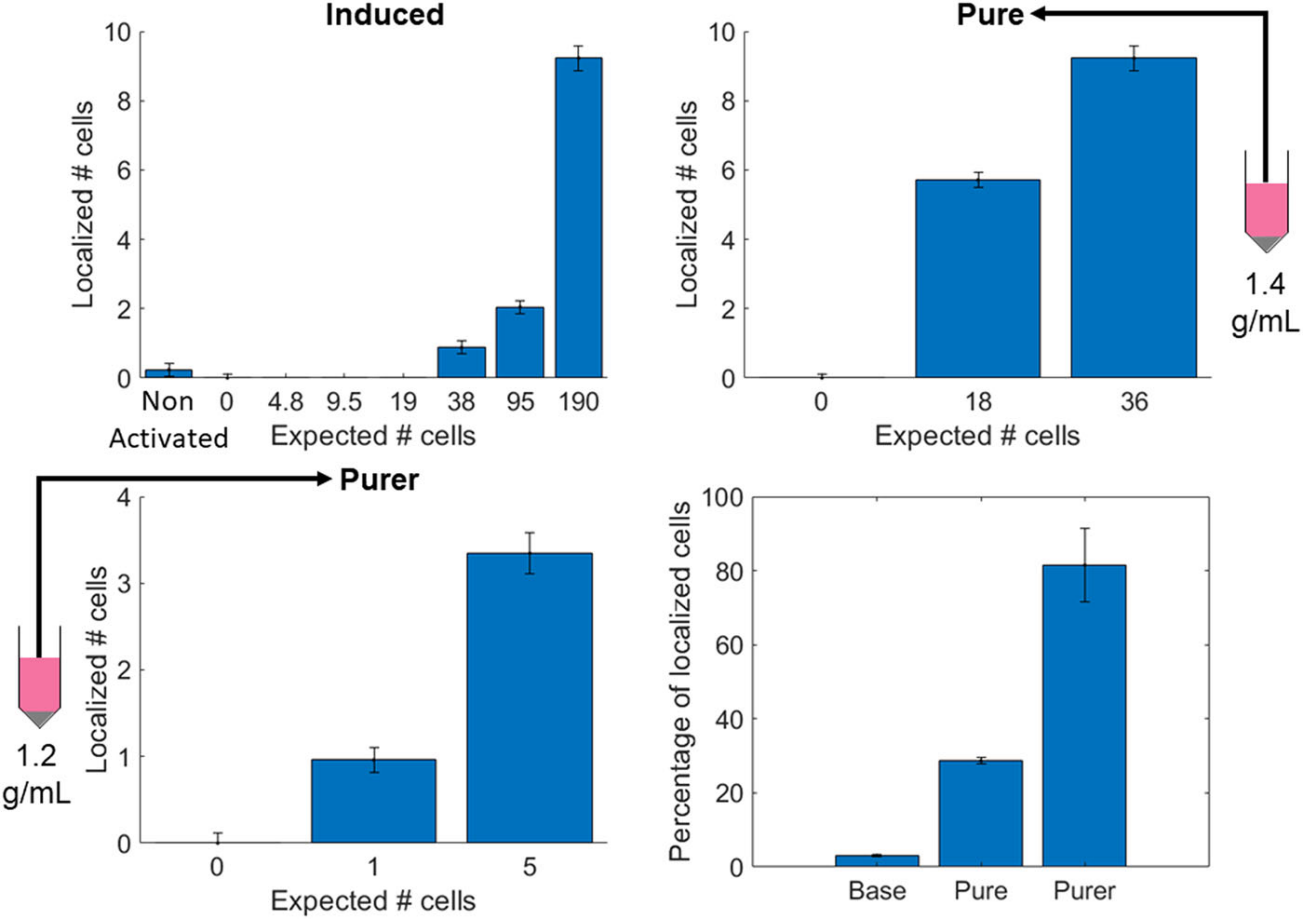
Concentration sweeps demonstrating the number of cell localisations with deactivation super-resolution imaging compared to the expected number of cells based on the cell concentration flowing at 3 levels of buoyancy purification, along with a summary figure (bottom right) showing the percentage of localised cells across the different concentrations for each purification method.

To test the sensitivity of DSR we conducted an experiment in which cells were flowed at varying of concentrations through the cross-tube phantom while imaging. We compared the number of cells localised against expected cell counts based on the volume of liquid being imaged and the concentration of cells as determined by haemocytometer cell counting. We then developed a buoyancy purification technique based on centrifugating GV-expressing cells in liquids of varying density (ranging from 1.2 to 1.4 g ml^−1^). Cells that express more GVs have a lower volume-averaged density and therefore float to the top in less-dense media. After separating cells using this method, we repeated cross-tube imaging at different levels of purity (Fig. 5).

**Fig. 6:**
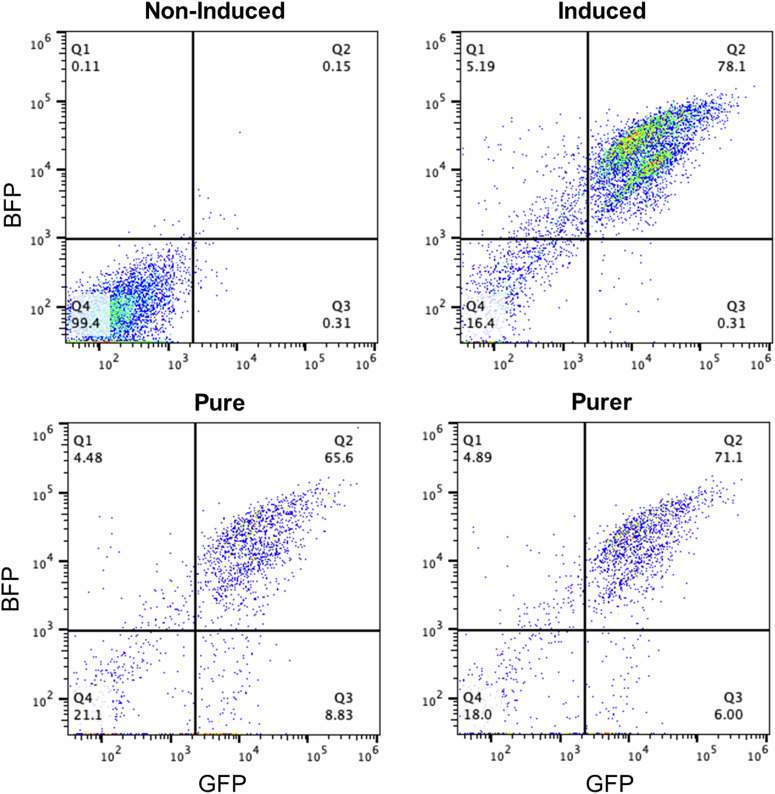
Flow cytometry showing GFP and BFP expression across induced, non-induced, and purified cells.

We found that for the base induced cells roughly 3% of the cells produced enough GVs to be individually localised, for the cells purified at 1.4 g ml^−1^ 29% could be localised, and for cells purified at 1.2 g ml^−1^ this value was 81%. Additionally, analysed samples using a flow cytometer to assess the proportion of cells expressing GFP and BFP across the different purification levels (Fig. 6). As expected, cells that were not induced with doxycycline were largely non-fluorescent in BFP and GFP, whereas the induced cells there is roughly a 78% double positive population. In the Pure and Purer buoyancy purified cell lines we see roughly 66% and 71% double-positive (BFP and GFP) populations, respectively. These results suggest that DSR imaging is a more effective and direct method to quantify GV expression levels in a cellular population than a fluorescence-based surrogate.

## Discussion

In this work we investigated the capability of the DSR technique, achieving a separation distance of 0.30 mm, which is a notable improvement over the traditional axial resolution limit without super resolution of 0.56 mm^27^. Additionally, it is notable that the FWHM and separation distances for the B-mode image are comparable, but this was not the case for the DSR method. The FWHM of the DSR method was found to be 0.21 mm, which is only marginally larger than the tube diameter of 0.2 mm, suggesting that the true FWHM of the imaging technique may be smaller than this experiment suggests, and therefore perhaps that the true separation distance of DSR is here overestimated.

It is worth noting that the cells frequency express multiple GVs. The agent being super-resolved in this work is not the GV directly but the cell containing the GVs, and the point spread function being imaged is likely contributed to by a cluster of GVs. Looking at the results of the buoyancy purification experiments, we find evidence that the DSR method is capable of imaging individual cells. First, we see secondary evidence in the fact that as the purification levels increase the percentage of cells that are localised relative to the number of expected cells increases dramatically. The primary evidence lies in the combination of the following two facts. First, we see almost no cells in our 0 cells controls. This rules out false positives and demonstrates that what we are localising are indeed cells. Second, in our most purified samples we see around 80% of the expected number of cells. As this number is significantly greater than 50% this rules out the possibility that we are visualising groups of multiple cells. This thereby demonstrates that the DSR technique is capable of imaging individual cells, and that the technique could be useful as a method to determine GV levels inside cells.

While the DSR pipeline proved capable of super-resolving individual cells, there are many stages of the pipeline where further optimisation may be possible. There are a large number of potential methods of separating GV signal from background, noise thresholding, cell isolating and centroid localisation. Future work will require further refinement of this technique, which may allow for further increases in the percentage of localised cells and a decrease in GV expression levels necessary for imaging.

Regarding the flow cytometry data, we find both that the percentage of cells that are GFP and BFP positive does not increase due to buoyancy purification, and also that the strength of the fluorescence is unchanged. This suggests that while GFP and BFP gating can indicate whether or not cells are producing GVs, outside of this binary there is no correlation between levels of fluorescence and levels of GV expression. This also implies that buoyancy filtration cannot be substituted by fluorescence-based means, and that fluorescence-based estimates of GV expression are insufficient, highlighting the importance of acoustic-based methods such as the one proposed. Genetic engineering efforts are ongoing to ensure more consistent expression of GVs across a cell population^28^.

In conclusion, we have demonstrated that DSR is an effective super-resolution technique in vitro, producing images with an ~4-fold improvement in resolution compared to conventional ultrasound imaging. DSR has proven capable of localising individual GV-expressing mammalian cells. Future studies are needed to demonstrate how this capability can be used super-resolve biological phenomena in vivo and beyond the vascular space in basic biology and potential clinical applications. In the meantime, in vitro cross-tube imaging provides a useful method to assess GV expression levels inside cells and evaluate the achievable resolution.

## Methods

### Cross tube phantom experiments

To evaluate the effectiveness of DSR, we used a cross tube phantom^24–26^ comprising of two thin-walled cellulose capillary tubes (Hemophan®, Membrana) with an internal diameter of 200 μm ± 15 μm, a wall thickness of 8 ± 1 μm in the dry state, and a length change under wet conditions of ±1%. We placed these two tubes close together in a cross formation, with cells pulled upwards by a syringe pump (Pump 33 DDS, Harvard Apparatus, Massachusetts, USA). This crossed shape allows for the measurement of the two tubes at a wide range of distances from each other, enabling the determination of the imaging resolution by identifying the point at which the tubes merge and become indistinguishable due to overlap in the point spread functions.

Imaging was performed using an L11-4v probe and a Verasonics Vantage 256 system, transmitting 3 cycle pulse trains with a 4 MHz centre frequency. The cells we used were MDA-MB-231-mARG_Ana_ cells as described in Hurt et al.^13^. In brief, we engineered MDA-MB-231 cells to constitutively co-express the plasmids shown in Fig. 3 (top) which enable expression of GVs derived from anabaena, GFP and BFP when doxycycline induced (1 μg/mL). We then purified these cells via fluorescent sorting for co-expression of GFP and BFP and grew them out over several passages.

### Buoyancy purification experiments

We buoyancy purified cells by adding density medium (OptiPrep Density Gradient Medium, SigmaAldrich, Missouri, USA) to the media, achieving final densities of 1.04 g/mL (pure) or 1.02 g/mL (purer) and then centrifuged (500 × *g*, 5 min). To further investiage this, we analysed the purified samples using a flow cytometer (Attune NxT Flow Cytometer, Thermo Fisher Scientific, USA). Examining GFP and BFP production levels after gating out cells using forward side scatter.

### Deactivation imaging

We generated a series of six high-pressure B-mode images at 200 frames per second using a 4 MHz central frequency and a 3-cycle pulse train length for each pulse-echo cycle. The ultrasound pulses had a peak positive pressure of 6.2 MPa, a peak negative pressure of 3.9 MPa and a mechanical index (MI) of 1.9, as measured by a 0.5 mm needle hydrophone (Precision Acoustics, UK). Using the Verasonics system, we transmitted 128 focused waves at the centre of the crossed tubes (18 mm distance) and scanned through the field of view to generate 128 beamformed ray-lines.

These parameters were chosen based on empirical experimentation and evidence from previous studies^22^. Higher pressures were used to enhance signal strength and improve imaging sensitivity while remaining within safety limits. Sensitivity was assessed using the SNR and CR metrics, with an emphasis on maximising the percentage of detectable cells, as shown in Fig. 5. While longer pulse train lengths improve signal strength and imaging sensitivity, excessive cycles can reduce the imaging resolution^22^. The DSR pipeline later helped improved this resolution. However, considering the diminishing returns on signal sensitivity at longer cycles^22^ and the need for sparse signals for effective SR, a 3-cycle pulse train was selected.

200 frames per second was the highest achievable framerate given the number of transmits and the imaging depth. Focussed waves were used in transmission to generate high pressures, and 128 foci were used to maximise the lateral resolution of the 128-element array. A focal distance of 18 mm was chosen as it aligned with the elevational focus of the L11-4v, further increasing the pressure.

During the first of the six pulse-echo cycles (here called the deactivation pulse), the protein shell of the GV fractures^22^, releasing the gas within. This gas then cavitates over the remaining cycles, emitting a strong acoustic signal before collapsing on a microsecond timescale^22,29^. In the subsequent pulse-echoes the GVs are no longer present, meaning the reflected signal represents all structures in the imaging field except for the GVs. By calculating the median image intensity of the five images following the deactivation pulse and subtracting this from the deactivation image, we obtain a deactivation image that isolates the signal from the GVs (Fig. 1).

### DSR pipeline

To super-localise the bubbles we implemented the processing pipeline illustrated in Fig. 2. After generating the deactivation image, we applied a noise threshold, followed by a peak-detecting adaptive threshold as described by Bradley et al.^30^ with a sensitivity of 0.1. The primary goal in setting these thresholds was to minimise false positive detections. As such the datasets containing 0 cells were processed first, and thresholds were adjusted accordingly. This needed to be balanced against the secondary goal of maximising the detection of the true positive cells.

With the noise successfully removed from the image we applied a normalised 2D cross-correlation with a 2D Gaussian, whose width was empirically derived to match the size of the point spread function. This process generated an image with maxima at locations where the deactivation image most closely resembled the point spread function. The point spread function was empirically determined by measuring the average FWHM of a series of sparse bright spots, presumed to be cells, using the low concentration experiments.

We threshold the correlated image and apply size gating to ensure that detected regions contain a pixel count consistent with expected sparse cell sizes. Thresholds and minimum size gating were again chosen to minimise false detections in the 0 cell datasets. Maximum size gating ensured that only sparse, individual cells were considered, as scarcity is essential for SR.

Finally, we estimated the locations of each super-resolved cell using the centroids of the thresholded regions. For display purposes we then convolved with a 2D Gaussian (FWHM 0.15 mm) to produce the final SR image.

## Data Availability

All data is available on request.
